# Common workflow language (CWL)-based software pipeline for *de novo* genome assembly from long- and short-read data

**DOI:** 10.1093/gigascience/giz014

**Published:** 2019-03-01

**Authors:** Pasi K Korhonen, Ross S Hall, Neil D Young, Robin B Gasser

**Affiliations:** Department of Veterinary Biosciences, Melbourne Veterinary School, The University of Melbourne, Parkville, Victoria 3010, Australia

**Keywords:** genome assembly, workflow language, workflow automation, repeatability

## Abstract

**Background:**

Here, we created an automated pipeline for the *de novo*assembly of genomes from Pacific Biosciences long-read and Illumina short-read data using common workflow language (CWL). To evaluate the performance of this pipeline, we assembled the nuclear genomes of the eukaryotes *Caenorhabditis elegans* (∼100 Mb), *Drosophila melanogaster* (∼138 Mb), and *Plasmodium falciparum* (∼23 Mb) directly from publicly accessible nucleotide sequence datasets and assessed the quality of the assemblies against curated reference genomes.

**Findings:**

We showed a dependency of the accuracy of assembly on sequencing technology and GC content and repeatedly achieved assemblies that meet the high standards set by the National Human Genome Research Institute, being applicable to gene prediction and subsequent genomic analyses.

**Conclusions:**

This CWL pipeline overcomes current challenges of achieving repeatability and reproducibility of assembly results and offers a platform for the re-use of the workflow and the integration of diverse datasets. This workflow is publicly available via GitHub (https://github.com/vetscience/Assemblosis) and is currently applicable to the assembly of haploid and diploid genomes of eukaryotes.

## Background

The assembly of genomes to chromosomal contiguity for many eukaryotic organisms has turned out to be a daunting task but has been achieved, for instance, for *Homo sapiens, Mus musculus, Caenorhabditis elegans, Drosophila melanogaster*, and *Plasmodium falciparum* [1–6]. The reference genomes of these organisms now meet the quality requirements set by the National Human Genome Research Institute (NHGRI-NIH) [7], namely, that the accuracy of the assembled nucleotides is at least 99.99% (≤1 nucleotide error over 10,000 bp), decontaminated contigs (each >30 kb) are ordered to form chromosomes, the sizes of gaps between any two contigs have been estimated, and the completeness of each chromosome is ≥95%.

For the first completed genome assemblies (i.e., *C. elegans* and *H. sapiens*), effective but costly and time-consuming bacterial artificial chromosome-based Sanger sequencing approaches were used [1, 3]. The use of less expensive, second-generation sequencing technologies [8, 9], such as Illumina [10], led to a rapid expansion in the number of draft genome assemblies for a range of metazoan organisms [11]. However, due to the inability to resolve repetitive DNA regions using short nucleotide read (50–300 bp) datasets [12], draft genomes are typically incomplete, fragmented, and contain mis-assembled regions, all of which constrain gene predictions and any subsequent genomic analyses [9, 13]. Nonetheless, novel draft genomes have opened up exciting new avenues for research on many non-model organisms, including parasites [14–20]. Some of these parasites cause neglected tropical diseases (NTDs), collectively representing a burden ≥1% of disability-adjusted life years per annum worldwide, with a related annual cost of anthelmintic treatment estimated at $3 billion [21]. In addition, resistance to anthelmintic drugs, used in mass drug administration, is a looming threat [22–25]. For these reasons, there is an imperative to advance genomic and systems biological research of these pathogens in order to gain a deep understanding of areas such as parasite biology, parasite-host interactions, disease, and drug resistance. The availability of high-quality genome assemblies is, thus, of utmost importance and could expedite the identification of novel drug targets and the design of advanced interventions (anthelmintics and vaccines) and diagnostic systems for the improved control of NTDs.

To enhance assembly quality, the use of long genomic reads (<100 kb in length) produced using third-generation sequencing technologies allows the resolution of long repeat regions and substantially reduces fragmentation [8]. With the use of scaffolding technologies, such as Hi-C [26] and BioNano [27, 28], the gap toward achieving high-quality de novo genome assemblies is closing [29]. The most prominent third-generation sequencing platforms currently available are the Pacific Biosciences (PacBio) single-molecule, real-time sequencer (RS) [30–32] and the *in silico* nanopore-based MinION and GridION systems from Oxford Nanopore [33]. The error rates in sequences generated using these technologies are ∼ 13% and 5%–40%, respectively [34, 35], and ∼15% for 1D and ∼5% for 1D^2^ for the latest 2016 Nanopore R9 chemistry, such that substantial sequencing depth is required to resolve sequencing errors [36]. Genomes assembled from sequence data from these platforms typically exhibit high numbers of indels. Depending on sequencing depth, it is common to employ accurate short-read data to validate or resolve inaccuracies in such genomes using a process referred to as “polishing” [29, 36, 37]. The quality and completeness of genome assemblies can be affected by quality and yield of DNA isolated from organisms, such as parasites, and challenges associated with extracting nucleic acids from them [38, 39]. DNA quantity is often limited because of the small size of some parasites and a need to isolate DNA from multiple organisms rather than one. There are often challenges in acquiring material from patients in distant locations, the cost of transport of such materials to a laboratory, and complications relating to microbial contamination, DNA degradation and nicking, co-purification of contaminating constituents, such as carbohydrates and lipids [38–40], and/or unique aspects, such as chromosomal diminution, in some parasites [41]. Clearly, the quality and amount of DNA have a major impact on completeness of a final genome assembly, irrespective of the sequencing technology employed.

A suitable computing environment and software tools are essential for producing a high-quality genome assembly. Such tools have dependencies on one another, particularly in terms of running order and software versions, and often require custom scripts for the integration of tools. Therefore, a substantial amount of time and effort is often required to complete a new assembly from scratch. Recently, issues surrounding the repeatability and reproducibility of results and reusability of datasets have been emphasized as being critical for scientific research [42–45], which have been neglected in some fields. Results are (i) repeatable, if the same findings are achieved multiple times using the same data [43]; (ii) reproducible, if the same findings are achieved multiple times using reproduced data [43]; and (iii) reusable, if new results are achieved using new data [42]. There is clear evidence that the repeatability of experiments that use software tools in published, peer-reviewed literature and the reusability of software for new experiments are challenging and/or error-prone [43, 46]; it is thus of prime importance to tackle these pertinent issues.

One possible approach would be to employ frameworks, such as SnakeMake [47], Ruffus/Rubra [48], Toil [49], and Rabix [50], or to use the common workflow language (CWL) [51] for workflows [52]. Each of these frameworks can be used to build bioinformatics pipelines, to execute complex tasks through the integration of software tools and the control of execution flow, in order to maximize the use of available computer resources and to ensure the repeatability of an experiment and reusability of a task. For instance, SnakeMake has been used in multiple workflows relating to RNA sequencing analyses [53], and Rubra is used in workflows such as RedDog [54] to infer single-nucleotide polymorphism (SNP) datasets derived from bacterial populations for subsequent phylogenetic analyses. By contrast, CWL defines a specification and offers a reference implementation, instead of providing a complete framework. The major advantage of CWL is its capacity to implement this specification for different computing environments and/or workflow frameworks, and CWL is already available in Toil and Rabix. To automate software installation, CWL supports “pull action” of Docker containers [46] and has beta-implementation for the integration of Bioconda (bioinformatics software package channel) [55]. Docker supports operating system virtualization [46] and has the capacity to form customized “containers” through the installation of particular software components. These containers can be deployed to different platforms, thereby conferring cross-platform portability [46]. Bioconda relies on the universal package manager Conda [56] to build binary software packages for Linux, MacOS, and Windows operating systems, to manage dependencies among software components within these packages, and to install packages locally into an isolated environment [55]. Although BioConda provides Docker containers for individual versions of a software tool to achieve high repeatability, built-in stochasticity of distinct versions has the potential to effect repeatability. CWL can use both Docker and Bioconda to install and run defined versions of software tools without manual intervention. Despite a growing interest in CWL, this framework has not yet gained the popularity that it deserves.

Here, employing CWL v1.0, we established an entirely novel, automated genome assembly pipeline [57] that integrates software tools and data from multiple sequencing platforms. This pipeline achieves repeatable and reproducible high-quality genome assemblies for metazoan organisms using PacBio sequence data, followed by “polishing” with Illumina short-read data. The pipeline resolves the dependencies among software packages via well-defined, versioned software packages that are automatically installed and executed at each step in the workflow, as required. This genome assembly pipeline should be broadly applicable in the biological and biomedical sciences.

## Results

### CWL assembly pipeline

**Figure 1:**
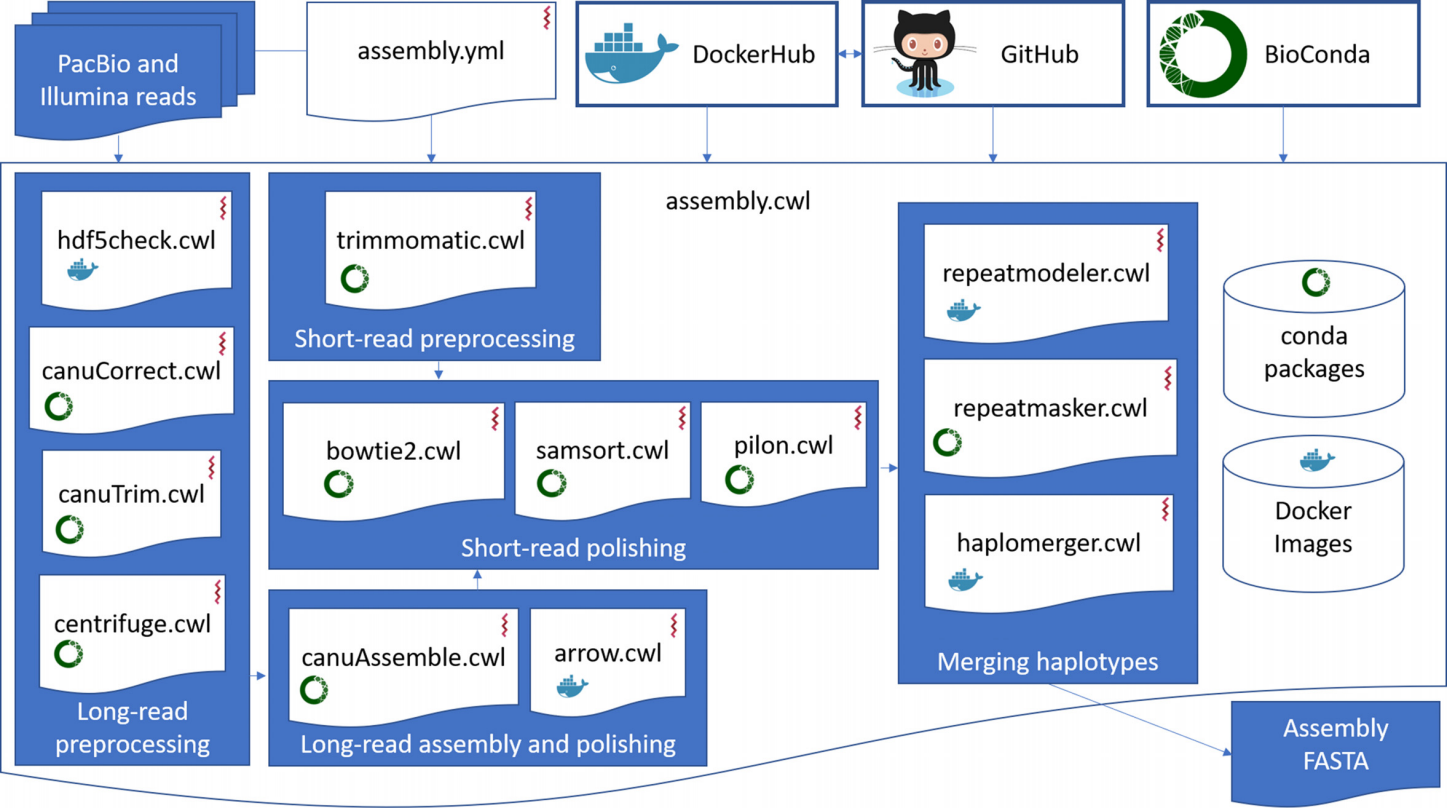
Diagram illustrates an automated common workflow language (CWL)-based genome assembly pipeline for PacBio long-read and Illumina short-read data. PacBio reads are first pre-processed and then used for assembly and long-read polishing. Illumina reads are cleaned and used to further polish the long-read assembly. Finally, haplotypes are merged in the repeat-masked, polished assembly. While the workflow is running, dependent software tools are automatically deployed from Bioconda package channel and DockerHub container repository. The code for the workflow and the Dockerfiles for the docker containers are stored in a GitHub code-repository.

The pipeline executes the programs integrated into the bioinformatics workflow (Fig. 1). First, PacBio reads from HDF5 formatted files that were converted to FASTA formatted files using the program Dextractor. These raw reads were then corrected using multiple rounds of read overlapping [58] and trimmed (e.g., removal of hairpin adapters and chimeric sequences) [36] using the program Canu. Subsequently, reads from potential contaminants (such as viruses, bacteria, and/or other microbes) were removed using the program Centrifuge, and remaining reads were assembled employing the program Canu. Using the program Arrow, PacBio raw reads were then employed to polish the assembly; further polishing was done with Illumina reads using the program Pilon. For polishing, Illumina reads were cleaned using the program Trimmomatic, mapped to the Arrow-polished assembly using the program Bowtie2, and sorted using the program SAMtools. For haplotype removal from the resultant assembly, custom repeat regions were inferred using the program RepeatModeler. The assembly was then masked employing inferred custom repeats, known transposons, and inferred tandem repeats using the program RepeatMasker. Finally, the program HaploMerger2 was used to identify and then remove the duplicated haplotypes from the masked Pilon-polished assembly, resulting in the final *de novo*-assembled diploid genome. Docker containers used in the pipeline were deposited to DockerHub [46] and automatically deployed using the software udocker. Required software tools were automatically fetched from Bioconda and installed into the target compute environment.

### Pipeline assemblies

Using the CWL assembly pipeline, the reference genomes of *C. elegans, D. melanogaster*, and *P. falciparum* were each re-assembled from publicly available PacBio and Illumina datasets (Table 1). Quality metrics were calculated for the resultant assemblies at each phase of the pipeline, i.e., Canu contigs, Arrow-polished contigs, Pilon-polished contigs, and haplo-merged contigs (Tables 2–4). For *P. falciparum* with haploid DNA [59], the Pilon-polished contigs represented the final assembly.

**Table 1:** Statistics for the PacBio long-read and Illumina short-read datasets and for reference genomes of *Caenorhabditis elegans, Drosophila melanogaster*, and *Plasmodium falciparum*^a^

Description	*Caenorhabditis elegans*	*Drosophila melanogaster*	*Plasmodium falciparum*
PacBio raw reads (bp)	4,726,985,993	15,733,529,928	5,246,949,826
read count; average length (bp)	411,459; 11,488	1,657,183; 9,494	515,155; 10,185
PacBio corrected reads (bp)	3,795,130,237	5,258,127,473	653,116,132
read count; average length (bp)	256,228; 14,812	279,988; 18,780	32,211; 20,276
PacBio trimmed reads (bp)	3,644,992,500	5,080,646,626	600,631,753
read count; average length (bp)	248,954; 14,641	271,623; 18,705	30,866; 19,459
PacBio contaminated reads (bp)	36,479,366	20,369	50,389
read count; average length (bp)	2,647; 13,781	1; 20,369	4; 12,597
PacBio decontaminated reads (bp)	3,608,513,134	5,080,626,257	600,581,364
read count; average length (bp)	246,307; 14,651	271,622; 18,705	30,862; 19,460
Illumina PE raw reads (bp)	24,028,252,320	42,492,715,000	61,074,625,500
read count; average length (bp)	200,235,436; 120	424,927,150; 100	244,298,502; 250
Illumina PE cleaned reads (bp)	16,914,423,470	28,126,765,439	13,370,453,180
read count; average length (bp)	66,608,171; 112	312,148,126; 90	87,161,538; 153
Sequencing depth for PacBio raw data	47	109	225
Sequencing depth for trimmed and decontaminated PacBio reads	36	35	26
Sequencing depth for Illumina raw reads	240	296	2,625
Sequencing depth for Illumina cleaned reads	169	196	575
Genome size (bp); sequence count	100,286,401; 7	137,567,484; 8	23,292,622; 14
Number of N nucleotides; gap count	0; 0	490,385; 268	0; 0
NG90 (bp); LG90	13,783,801; 6	23,513,712; 5	1,067,971; 12
NG50 (bp); LG50	17,493,829; 3	25,286,936; 3	1,687,656; 5
GC-content (%)	35.44	42.08	19.34
Complete BUSCO ortholog count	968	1,653	148
Complete single-copy BUSCO ortholog count	962	1,641	148
Complete duplicated BUSCO ortholog count	6	12	0
Fragmented BUSCO ortholog count	8	3	1
Missing BUSCO ortholog count	6	2	66
Expected BUSCO ortholog count	982	1,658	215
Length of coding sequences in reference (bp)	24,681,654	21,683,562	12,552,304
Length of non-coding sequences in reference (bp)	75,604,747	115,883,922	10,740,318
Number of reference coding sequences	20,081	13,911	5,515
Estimated repeat content (%); interspersed repeats (%)	18.95;18.20	20.52;19.04	21.84;4.41

^a^ *Caenorhabditis elegans* (National Center for Biotechnology Information [NCBI] accession identifier SRR2598966; URL [60]), *Drosophila melanogaster* [61] (NCBI Sequence Read Archive (SRA) accession identifiers SRX499318 and SRR1211256), and *Plasmodium falciparum* (NCBI SRA accession identifiers SRR3194817–25 and ERR862169–70) [59].

**Table 2:** Metrics for the *pipeline* assemblies of the *Caenorhabditis elegans* genome against the reference assembly for this species

Metric	Canu contigs	Arrow-polished contigs	Pilon-polished contigs	HaploMerger2- merged contigs
Genome size (bp)	104,147,712	104,179,922	104,199,510	102,615,360
Sequence count	100	100	100	54
Quast genome fraction (%)	97.29	97.64	97.56	97.00
Quast aligned length (bp)	98,056,933	98,420,852	98,371,646	97,651,504
Number of Ns (bp); gap count	0;0	0;0	0;0	0;0
N(G)90 (bp); L(G)90	973,097;34	973,604;34	973,839;34	1,058,765;27
N(G)50 (bp); L(G)50	2,859,879;11	2,860,369;11	2,860,908;11	4,165,666;9
GC content (%)	35.44	35.45	35.45	35.44
Repeat content (%); interspersed repeats (%)	-	-	20.64;19.33	20.41;19.17
Longest sequence (bp)	7,357,248	7,359,834	7,361,197	11,799,614
Shortest sequence (bp)	8,435	8,435	8,429	16,463
Quast number of translocations; relocations; inversions	1;41;14	1;36;14	1;38;15	5;40;13
Quast number of local mis-assemblies	891	709	722	696
Quast duplication ratio	1.005	1.005	1.005	1.004
Quast mis-matches	15,037	15,355	14,414	13,869
Quast indels (≤5 bp; >5 bp)	41,302;698	21,859;811	5,397;764	5,325;743
Quast indels length	58,771	40,680	23,336	22,772
Quast mis-matches; indels per 100 kbp	15.41;43.04	15.68;23.15	14.73;6.3	14.26;6.24
GAGE missing reference bases (nt; %)	86,628;0.09	77,203;0.08	76,194;0.08	292,272;0.29
GAGE missing assembly bases (nt; %)	464,022;0.45	582,816;0.56	548,487;0.53	457,713;0.45
GAGE duplicated reference bases	4,962,481	4,775,862	4,834,860	3,510,166
GAGE compressed reference bases	596,736	586,626	595,695	712,344
GAGE average identity (%)	99.92	99.94	99.96	99.96
GAGE nucleotide mis-matches	10,407	9,883	9,921	9,964
GAGE indels (≤5 bp; >5 bp)	49,111;529	24,590;526	5,866;527	6,076;528
GAGE number of translocations; relocations; inversions	32;270;129	35;124;300	29;129;300	42;132;290
Complete single-copy; duplicated BUSCO ortholog count	948;6	963;6	964;7	964;6
Fragmented; missing BUSCO ortholog count	21;7	10;3	8;3	8;4
Number of nucleotide mis-matches in; outside CDSs	1,209;13,828	1,156;14,199	1,154;13,260	1,222;12,647
Number of indels in; outside CDSs	3,580;38,357	1,104;21,499	177;5,889	149;5,825
Number of affected mRNAs; proteins	2,877;2,858	969;948	154;131	144;121
Number of non-synonymous; synonymous mutations	483;553	515;590	443;551	485;579
Number of in-frame indels	101	49	48	61
Combined accuracy of mis-matches and indels in coding regions (%)	99.981	99.991	99.995	99.994
Combined accuracy of mis-matches and indels in non-coding regions (%)	99.789	99.855	99.922	99.925

**Table 3:** Metrics for *pipeline* assemblies of the *Drosophila melanogaster* genome against the reference assembly for this species

Metrics	Canu contigs	Arrow-polished contigs	Pilon-polished contigs	HaploMerger2- merged contigs
Genome size (bp)	157,857,743	157,985,917	157,986,071	129,695,906
Sequence count	439	439	439	61
Quast genome fraction (%)	97.907	98.1	98.095	91.514
Quast aligned length (bp)	138,910,049	139,294,859	139,287,556	126,646,721
Number of Ns (bp); gap count	0;0	0;0	0;0	0;0
N90 (bp); L90	138,987;78	139,113;78	139,125;78	1,615,500;10
N50 (bp); L50	10,648,637;6	10,656,889;6	10,656,888;6	13,348,143;4
NG90 (bp); LG90	105,872;95	104,289;96	104,289;96	1,615,500;10
NG50 (bp); LG50	8,532,606;7	8,534,347;7	8,534,351;7	16,059,280;3
GC content (%)	41.68	41.68	41.68	42.17
Repeat content (%); interspersed repeats (%)	-	-	30.15;28.84	16.54;14.59
Longest sequence (bp)	21,669,562	21,676,918	21,676,919	25,791,812
Shortest sequence (bp)	2,688	2,688	2,688	7,073
Quast number of translocations; relocations; inversions	74;60;2	74;60;2	74;60;2	39;24;0
Quast number of local mis-assemblies	610	652	645	313
Quast duplication ratio	1.031	1.032	1.032	1.006
Quast mis-matches	8,441	6,256	6,590	4,909
Quast indels (≤5 bp; >5 bp)	41,716;402	8,399;390	8,480;390	7,222;279
Quast indels length	51,453	16,762	16,911	12,871
Quast mis-matches; indels per 100 kbp	6.27;31.28	4.64;6.51	4.88;6.57	3.9;5.96
GAGE missing reference bases (nt; %)	643,319;0.47	644,217;0.47	646,300;0.47	4,913,341;3.57
GAGE missing assembly bases (nt; %)	3,608,718;2.29	3,655,639;2.31	3,655,348;2.31	522,589;0.40
GAGE duplicated reference bases	23,437,831	23,161,535	23,181,331	3,623,824
GAGE compressed reference bases	1,919,237	1,778,270	1,783,342	7,621,896
GAGE average identity (%)	99.95	99.98	99.98	99.98
GAGE nucleotide mis-matches	7,292	5,657	6,622	5,459
GAGE indels (≤5 bp; >5 bp)	49,597;273	9,393;245	9,506;245	8,825;213
GAGE number of translocations; relocations; inversions	14;267;73	15;306;75	15;306;69	96;235;96
Complete single-copy; duplicated BUSCO ortholog count	1,618;19	1,634;19	1,634;19	1,639;11
Fragmented; missing BUSCO ortholog count	17;4	2;3	2;3	2;6
Number of nucleotide differences in; outside CDSs	1,697;6,744	1,586;4,670	1,502;5,088	1,584;3,325
Number of indels in; outside CDSs	4,953;37,143	157;8,576	158;8,656	194;7,272
Number of affected mRNAs; proteins	2,660;2,640	123;105	128;109	133;120
Number of non-synonymous; synonymous mutations	687;650	586;612	575;539	590;604
Number of in-frame indels	94	52	48	42
Combined accuracy of mis-matches and indels in coding regions (%)	99.969	99.992	99.992	99.992
Combined accuracy of mis-matches and indels in non-coding regions (%)	99.798	99.939	99.937	99.951

**Table 4a:** Metrics for *pipeline* assemblies of the *Plasmodium falciparum* genome against the reference assembly for this species

Metrics	Canu contigs	Arrow-polished contigs	Pilon-polished contigs
Genome size (bp) (apicoplast removed)	23,328,599	23,350,837	23,350,454
Sequence count (apicoplast removed)	14	14	14
Apicoplast genome (bp)*	-	-	34,274
Quast genome fraction (%)	99.62	99.529	99.648
Quast aligned length (bp)	23,252,840	23,248,663	23,276,411
Number of Ns (bp); gap count	0;0	0;0	0;0
N(G)90 (bp); L(G)90	1,058,353;12	1,059,223;12	1,059,208;12
N(G)50 (bp); L(G)50	1,709,389;5	1,711,020;5	1,710,975;5
GC content (%)	19.34	19.33	19.33
Repeat content (%); interspersed repeats (%)	-	-	22.45; 6.78
Longest sequence (bp)	3,291,378	3,294,104	3,294,056
Shortest sequence (bp)	642,032	642,892	642,874
Quast number of translocations; relocations; inversions	0;2;0	0;2;0	0;2;0
Quast number of local mis-assemblies	43	47	47
Quast duplication ratio	1.002	1.003	1.003
Quast mis-matches	2,237	1,242	1,503
Quast indels (≤5 bp; >5 bp)	14,422;174	9,241;168	8,783;180
Quast indels length	21,049	14,430	13,977
Quast mis-matches; indels per 100 kbp	9.64;62.9	5.36;40.59	6.48;38.62
GAGE missing reference bases (nt; %)	15,710;0.07	15,198;0.07	15,333;0.07
GAGE missing assembly bases (nt; %)	12,584;0.05	12,774;0.05	12,658;0.05
GAGE duplicated reference bases	112,885	281,583	193,259
GAGE compressed reference bases	122,934	89,625	89,404
GAGE average identity (%)	99.88	99.93	99.93
GAGE nucleotide mis-matches	3,094	1,107	1,281
GAGE indels (≤5 bp: >5 bp)	19,815;156	11,923;128	11,450;131
GAGE number of translocations; relocations; inversions	14;12;9	35;12;10	34;12;11
Complete single-copy; duplicated BUSCO ortholog count	147;0	148;0	148;0
Fragmented; missing BUSCO ortholog count	1;67	1;66	1;66
Number of nucleotide mis-matches in; outside CDSs	420;1,817	356;886	348;1,155
Number of indels in; outside CDSs	1,009;13,577	573;8,826	486;8,466
Number of affected CDSs	732	430	369
Number of affected mRNAs; proteins	711;704	420;418	362;360
Number of all anomalies	15,394	9,712	9,621
Number of non-synonymous; synonymous mutations	233;187	189;167	179;169
Number of in-frame indels	131	84	61
Combined accuracy of mis-matches and indels in coding regions (%)	99.979	99.989	99.988
Combined accuracy of mis-matches and indels in non-coding regions (%)	99.875	99.921	99.922

^*^Circlator [67] was used to establish the size of apicoplast genome.

**Table 4b:** Metrics for unpolished and polished Vembar assemblies of the *Plasmodium falciparum* genome against the reference assembly

Metrics	Vembar assembly	Arrow-polished Vembar assembly	Pilon-polished Vembar assembly
Genome size (bp) (apicoplast removed)	23,556,156	23,527,671	23,548,582
Sequence count (apicoplast removed)	20	20	20
Quast genome fraction (%)	98.965	99.214	98.526
Quast aligned length (bp)	23,203,419	23,233,198	23,093,770
Number of Ns (bp); gap count	0;0	0;0	0;0
N(G)90 (bp); L(G)90	1,063,883;12	1,062,674;12	1,063,566;12
N(G)50 (bp); L(G)50	1,712,288;5	1,710,421;5	1,711,745;5
GC content (%)	19.37	19.4	19.37
Longest sequence (bp)	3,299,835	3,294,973	3,298,759
Shortest sequence (bp)	24,138	24,220	24,138
Quast number of translocations; relocations; inversions	0;3;0	0;2;0	0;3;0
Quast number of local mis-assemblies	46	43	45
Quast duplication ratio	1.007	1.005	1.006
Quast mis-matches	1,233	1,396	1,365
Quast indels (≤5 bp; >5 bp)	31,261;546	9,391;213	23,638;533
Quast indels length	52,962	15,731	44,775
Quast mis-matches; indels per 100 kbp	5.35;137.98	6.04;41.56	5.95;105.32
GAGE missing reference bases (nt; %)	9,435;0.04	3,215;0.01	9,185;0.04
GAGE missing assembly bases (nt; %)	48,492;0.21	101,507;0.43	48,137;0.20
GAGE duplicated reference bases	239,012	330,347	219,507
GAGE compressed reference bases	146,954	97,331	172,885
GAGE average identity (%)	99.76	99.92	99.79
GAGE nucleotide mis-matches	2,502	1,197	2,010
GAGE indels (≤5 bp: >5 bp)	47,266;477	13,187;161	38,900;478
GAGE number of translocations; relocations; inversions	69;29;11	39;20;9	61;23;10
Complete single-copy; duplicated BUSCO ortholog count	141;0	146;0	146;0
Fragmented; missing BUSCO ortholog count	1;73	1;68	1;68
Number of nucleotide mis-matches in; outside CDSs	442;791	383;1,013	449;916
Number of indels in; outside CDSs	4,172;27,619	669;8,925	1,748;22,403
Number of affected CDSs	2,099	465	1,040
Number of affected mRNAs; proteins	1,949;1,947	457;454	1,001;999
Number of all anomalies	28,410	9,938	23,319
Number of non-synonymous; synonymous mutations	252;190	209;174	252;197
Number of in-frame indels	268	95	169
Combined accuracy of mis-matches and indels in coding regions (%)	99.978	99.988	99.984
Combined accuracy of mis-matches and indels in non-coding regions (%)	99.769	99.919	99.810

**Table 4c:** Metrics between the Vembar and *pipeline* assemblies of the *Plasmodium falciparum* genome

Metrics	Pilon-polished contigs vs. Vembar assembly	Arrow-polished contigs vs. Vembar assembly	Arrow-polished Vembar assembly vs. Vembar assembly	Arrow-polished Vembar assembly vs. Arrow-polished contigs
Genome size (bp)	23,350,454	23,350,837	23,527,671	23,350,837
Sequence count	14	14	20	14
Quast genome fraction (%)	99.196	99.196	99.638	99.206
Quast aligned length (bp)	23,331,625	23,332,007	23,455,145	23,342,276
Number of Ns (bp); gap count	0;0	0;0	0;0	0;0
N(G)90 (bp); L(G)90	1,059,208;12	1,059,223;12	1,062,674;12	1,059,223;12
N(G)50 (bp); L(G)50	1,710,975;5	1,711,020;5	1,710,421;5	1,711,020;5
GC content (%)	19.33	19.33	19.4	19.33
Longest sequence (bp)	3,294,056	3,294,104	3,294,973	3,294,104
Shortest sequence (bp)	642,874	642,892	24,220	642,892
Quast number of translocation; relocation; inversions	2;4;0	1;0;0	0;0;0	1;1;0
Quast number of local mis-assemblies	8	9	7	3
Quast duplication ratio	0.999	0.999	1	1
Quast mis-matches	443	458	645	368
Quast indels (≤5 bp; >5 bp)	28,490;336	28,437;338	27,555;314	3,901;154
Quast indels length	41,790	41,736	39,998	7,753
Quast mis-matches; indels per 100 kbp	2.09;122.05	1.96;123.15	2.75;118.74	1.58;17.37
GAGE missing reference bases (nt/%)	45,726/0.19	45,177/0.19	3,275/0.01	40,737/0.17
GAGE missing assembly bases (nt/%)	3,742/0.02	3,524/0.02	3,191/0.01	1,022/0.00
GAGE duplicated reference bases	30,238	29,012	122,706	41,521
GAGE compressed reference bases	213,158	200,144	120,450	782,798
GAGE average identity (%)	99.81	99.81	99.82	99.97
GAGE nucleotide mis-matches	399	414	694	180
GAGE indels (≤5 bp; >5 bp)	39,377;213	39,755;212	38,586;183	5,923;43
GAGE number of translocation; relocations; inversions	49;20;1	46;16;1	32;15;0	35;8;2
Complete BUSCOs	148	148	146	148
Complete single-copy; duplicated BUSCO ortholog count	148;0	148;0	146;0	148;0
Fragmented; missing BUSCO ortholog count	1;66	1;66	1;68	1;66

### Completeness and contiguity

The final assembly for *P. falciparum* (23.4 Mb; GC content of 19.33%; no gaps and no unresolved nucleotides) represented complete chromosomes (n = 14) and a complete apicoplast genome (Table 4a). When aligned to the reference (23.3 Mb; GC content of 19.34%; no gaps and no unresolved nucleotides), the assembly had 15.3 kb, 193.3 kb, and 89.4 kb of “missing, duplicated, and compressed reference bases,” respectively (Table 4a). In terms of contiguity and completeness, the Arrow-polished assembly (23.4 Mb) was no different from the Pilon-polished one (Table 4a).

For *C. elegans*, the haplo-merged assembly (102.6 Mb; 54 contigs; NG50 of 4.2 Mb; LG50 of 9; LG90 of 27; GC content of 35.4%; no gaps and no unresolved nucleotides) was slightly larger than the reference (100.3 Mb; 7 chromosomes; no gaps and no unresolved nucleotides), the longest contig being 11.8 Mb (Table 2). Reference-aligned contigs had 292 kb, 3.5 Mb, and 712 kb of missing, duplicated, and compressed reference bases, respectively (Table 2). The Arrow- and Pilon-polished assemblies (104.2 Mb; 100 contigs; NG50 of 2.9 Mb; LG50 of 11; LG90 of 34) were more fragmented than the haplo-merged one, and had 76–77 kb, 4.8 Mb, and 587–596 kb of missing, duplicated, and compressed reference bases, respectively (Table 2). No mitochondrial genome was detected.

The haplo-merged assembly of *D. melanogaster* resulted in 61 contigs (N50 = 13.3 Mb; L50 = 4; L90 = 10; GC content of 42.2%; no gaps or unknown nucleotides) and was markedly smaller (129.7 Mb) than the reference genome (137.6 Mb; 7 chromosomes; 268 gaps and 490,385 unresolved nucleotides), with 4.9 Mb, 3.6 Mb, and 7.6 Mb of missing, duplicated, and compressed reference bases, respectively (Table 3). Both the Arrow- and Pilon-polished assemblies (158.0 Mb; 439 contigs; N50 of 10.7 Mb) had 644–646 kb, 23.2 Mb, and 1.8 Mb of missing, duplicated, and compressed reference bases, respectively, and were much larger and more fragmented than the reference assembly (Table 3).

The Benchmarking Universal Single-Copy Orthologs (BUSCO) results for *P. falciparum* (149 detected orthologs of a total of 216), *C. elegans* (978 of 982), and *D. melanogaster* (1,652 of 1,658) were very similar to those of their reference sequences (i.e., 149 of 216, 976 of 978, and 1,656 of 1,658, respectively; Tables 2, 3, and 4a). In comparison to pure Canu assemblies, Arrow mpolishing increased the number of complete BUSCO orthologs from 147 to 148, 952 to 969, and 1,637 to 1,653, respectively, and reduced the fragmented BUSCO orthologs in *C. elegans* from 21 to 10 and *D. melanogaster* from 17 to 2 (Tables 2–4). Pilon polishing did not change the total number of BUSCO orthologs detected but did reduce the number of fragmented orthologs by two for *C. elegans* (Tables 2, 3, and 4a).

### Accuracy

For *P. falciparum*, Quast metrics for the Pilon-polished assembly (nucleotide identity: 99.93%; repeat content: 22.45%, including interspersed repeats: 6.78%) indicated a modest number of mis-assemblies consisting of two relocations; together, 47 local mis-assemblies, 180 large indels, 8,783 small indels, and 1,503 nucleotide mis-matches (Table 4a). In total, 362 mRNAs were predicted to harbor 486 indels and 348 nucleotide differences (179 non-synonymous) in coding regions (12,552,304 bp), inferred to result in a share of 6.5% (360 of 5,515) mutated proteins (Table 4a). Non-coding regions represented by 10,740,318 bp had 8,466 indels and 1,155 nucleotide mis-matches (Table 4a). Arrow polishing with a coverage of 225x PacBio raw data decreased the number of indels in the Canu assembly from 14,596 to 9,409 and nucleotide mis-matches from 2,237 to 1,242 (Table 4a). Pilon polishing (coverage 575x cleaned Illumina reads) had only a minor positive effect on these results, i.e., indels decreased to 8,963, mis-matches increased to 1,503, and proteins predicted to be mutated decreased from 418 to 360 (Table 4a). Using the Pilon-polished assembly, results achieved for Quast and Genome Assembly Gold-Standard Evaluation (GAGE) (translocations [n = 34], relocations [n = 12], inversions [n = 11], 131 large indels, 11,450 small indels, and 1,281 nucleotide differences) were similar (cf. Table 4a).

For *C. elegans*, the haplo-merged assembly (identity: 99.96%; repeat content: 20.41%, including interspersed repeats: 19.17%) resulted in 561 mis-assemblies (5 translocation, 40 relocations, and 13 inversions), 696 local mis-assemblies, 743 large indels, 5,325 small indels, and 13,869 nucleotide mis-matches (Table 2). In coding regions (24,681,654 bp), there were 149 indels and 1,222 nucleotide mis-matches (485 non-synonymous) that were inferred to affect 144 mRNAs and to alter a share of 0.60% (121 of 20,081) proteins, whereas non-coding regions (75,604,747 bp) had 5,825 indels and 12,647 nucleotide mis-matches (Table 2). Arrow polishing with PacBio reads at a coverage of 47x resulted in a substantial reduction in the number of indels (42,000 to 22,670) and a minor increase in nucleotide differences (15,037 to 15,355) (Table 2). Pilon polishing (coverage: 169x of cleaned Illumina reads) substantially reduced further the number of indels to 6,161 and slightly reduced the nucleotide mis-matches to 14,414, reducing the number of proteins predicted to be mutated from 948 to 131 (Table 2). GAGE metrics for the haplo-merged assembly differed, with 42 translocations, 132 relocations, 290 inversions, 528 large indels,,076 small indels, and 9,964 nucleotide mis-matches recorded (Table 2).

For *D. melanogaster*, the haplo-merged assembly (identity: 99.98%; repeat content: 16.54% including interspersed repeats: 14.59%) had 63 mis-assemblies (39 translocations, 24 relocations, and no inversions), 313 local mis-assemblies, 279 large indels, 7,222 small indels, and 4,909 nucleotide mis-matches (Table 3). In coding regions (21,683,562 bp), there were 194 indels and 1,584 nucleotide mis-matches (590 non-synonymous), inferred to affect the 133 mRNA sequences, resulting in share of 0.86% (120/13,911) altered protein sequences (Table 3). In non-coding regions (115,883,922 bp), 7,272 indels and 3,325 nucleotide mis-matches were detected. Arrow polishing with PacBio reads (109x coverage) largely reduced the number of indels from 42,118 to 8,789 and nucleotide mis-matches from 8,441 to 6,256 (Table 3). Pilon polishing slightly increased the number of indels to 8,870, of mis-matches to 6,590, and of altered protein sequences from 105 to 109 (Table 3). GAGE metrics of the haplo-merged assembly resulted in 96 translocations, 235 relocations, 96 inversions, 213 large indels, 8,825 small indels, and 5,459 mis-matches (Table 3).

In the Arrow-polished *pipeline* assembly for *P. falciparum*, it was 18.0-fold more likely to observe indels in non-coding (8,826 indels/10 ,740,318 bp) than in coding regions (573 indels/12,552,304 bp) (Tables 1 and 4a). For *D. melanogaster*, this likelihood was 10.2-fold (8,576 indels/115,883,922 bp in non-coding vs 157 indels/21,683,562 bp in coding regions) and 6.4-fold for *C. elegans* (21,499 indels/75,604,747 bp in non-coding vs 1,104 indels/24,681,654 bp in coding regions) (Tables 1–3). For Pilon-polished *pipeline* assemblies, the likelihoods were 20.4, 10.3, and 10.9, respectively.

### Vembar assembly for *P. falciparum*

When compared with the reference assembly, the Vembar assembly resulted in 1,233 nucleotide mis-matches, 546 large indels, and 31,261 small indels (Table 4b). For the Arrow-polished Vembar assembly, the number of nucleotide differences increased slightly (n = 1,396), but the number of large (n = 213) and small (n = 9,391) indels was substantially reduced (Table 4b). The comparison of the Arrow-polished *pipeline* assembly to the Vembar assembly resulted in a modest number of nucleotide mis-matches (n = 458 bp), but in a high number of large (n = 338) and small (n = 28,473) indels (Table 4c). For the Pilon-polished *pipeline* assembly, the numbers were similar (n = 443 mis-matches; n = 336 large indels; n = 28,490 small indels) when compared with the Vembar assembly (Table 4c). However, the numbers of nucleotide differences (n = 368) and large (n = 154) and small (n = 3901) indels were small when the Arrow-polished Vembar assembly and the Arrow-polished *pipeline* assembly were compared (Table 4c). Both the Vembar assembly and Arrow-polished *pipeline* assembly shared 8,947 indels and 2,007 nucleotide differences in the same locations in the reference genome. For the Vembar assembly, it was 7.7-fold more likely for indels to be observed in non-coding (27,619 indels/10,987,349 bp) than in coding regions (4,172 indels/12,282,956 bp) (Tables 1 and 4a). The numbers of BUSCO orthologs detected were 142, 147, and 147 for the Vembar, Arrow-polished, and Pilon-polished Vembar assemblies, respectively (Table 4b).

### Indel correlations

**Figure 2:**
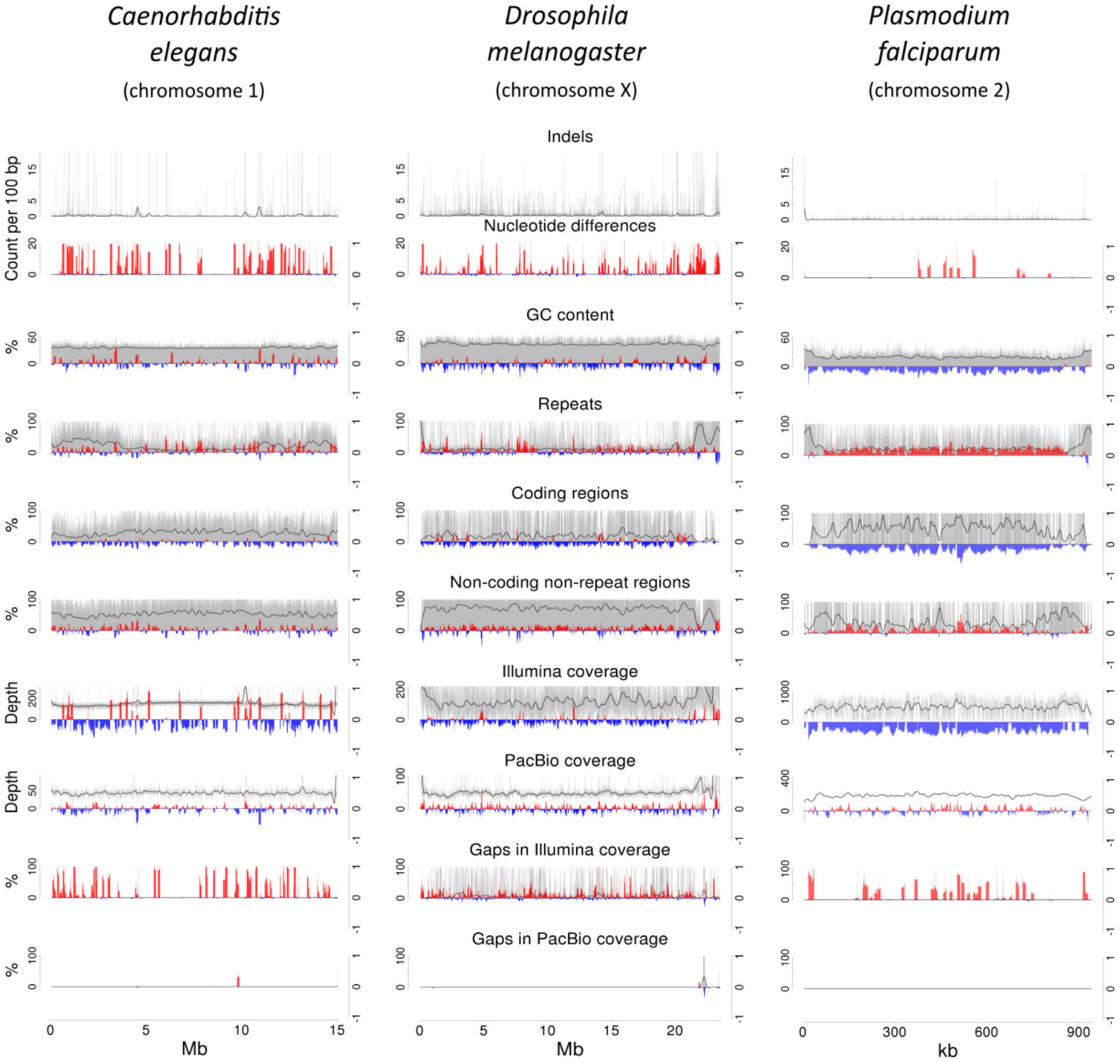
Correlation diagrams of indels are illustrated for one chromosome of each reference genome reassembled. The columns represent *Caenorhabditis elegans, Drosophila melanogaster*, and *Plasmodium falciparum*, from left to right. The *y*-axis on left side represents the data to correlate with indels (gray bars and smoothened black line), whereas red bars and blue bars on the right side represent positive and negative correlations, respectively. Clearly, the regions around indels correlate with those around nucleotide differences, repeat regions, non-coding non-repeat regions, and gaps in Illumina coverage. In contrast, regions around GC content, coding regions, and Illumina coverage correlate negatively to those around indels. As expected, due to lack of context bias, PacBio coverage does not show clear correlation to indels and have only few low coverage regions in these chromosomes. The correlation patterns for *C. elegans* and *D. melanogaster* follow those of *P. falciparum*, although they are not as conspicuous.

For *P. falciparum*, the genomic locations with indels correlated positively with positions of nucleotide differences, repeat regions, and gaps in mapping coverage and correlated negatively with coding regions, GC content, and Illumina-mapping coverage (Fig. 2). Although not as pronounced, a similar pattern was observed in both *C. elegans* and *D. melanogaster* (Fig. 2). None of the assemblies showed a clear distinction in correlation between PacBio sequencing depth and coding and/or repeat regions (Fig. 2). Telomeric regions, being at the ends of the chromosomes of *P. falciparum*, were clearly visible based on an abundance of repeats and a lack of coding sequences (Fig. 2).

## Discussion

In the present study, we demonstrate unequivocally that CWL is a language to clearly describe a workflow and develop a fully automated pipeline with capacities to parallelize its execution, to define dependencies to the order of execution, and to automatically install versioned software packages. Therefore, CWL offers a practical and convenient way for researchers to obtain repeatable and reproducible results from bioinformatics experiments for subsequent scientific publications. This language is highly suited to different compute environments for integration, the reuse of diverse datasets, and repeating or reproducing results reported from previous experiments (using CWL) published in the peer-reviewed literature. Current reference implementation of CWL does not scale to distributed compute systems but is usable on servers configured with multiple central processing units (CPUs). For the present assembly workflow, the use of software tools directly from Bioconda was preferred [62], and Docker containers were only created for custom scripts or if a tool was not available in Bioconda or dysfunctional. For instance, it was not possible to use RepeatModeler via Bioconda because the latest RepeatLibrary from RepBase could not be installed in that version. The integration of RepeatModeler with a Docker container resolved this issue. Thus, CWL allows an efficient integration of alternative tools and extensions, such as assemblers and new scaffolding tools.

Despite the successful creation of the present assembly workflow, CWL v1.0 has some limitations. The essential feature of container integration currently supports only Docker containers and, thus, can pose a serious security risk in a multi-user computing environment, such as high-performance computing (HPC) systems [63–65]. The container processes are spawned from a root-owned Docker daemon and, consequently, executed as a root, thus escaping policies to the privileged usage of resources and controls [64, 65], which may lead to “container escape attacks” [63]. For example, knowing that Docker daemon communicates either using a Unix- or TCP-socket and that the Unix socket typically has root: docker (user: group) rights, users who belong to the docker-group are granted root rights to resources such as file systems, communication protocols, and mounting, thereby exposing the environment to malicious and/or accidental mis-uses [63]. The possible case of daemon communicating via a TCP socket would allow misuse from outside of the server through an internet connection, if not appropriately configured [63]. The distribution of Docker images, for instance, from DockerHub, has the potential to lead to the distribution of malicious Dockerfiles through a compromised GitHub account [63]. The latter issue can be prevented by uploading docker images directly to DockerHub or by disabling the update-link between GitHub and DockerHub. CWL implementation addresses the security issue related to root rights by enforcing the user and group identifiers to those of the current user in Docker execution. However, a security risk still remains, because Docker containers can be used in non-CWL contexts and, therefore, should not be installed into a multi-user HPC environment. This security issue can be addressed in CWL by extending support to containers, such as the open source effort called Singularity [65], or by using an alternative Docker implementation, such as rootless udocker, which was shown to be successful in the present study.

In addition to security aspects, minor issues relating to the use of CWL were encountered. For instance, CWL enforces read-only access to the file system inside a Docker container, thereby creating unnecessary complexity when using some tools, such as SmrtLink. Specifically, in SmrtLink, the creation of reference genomes in the file system is hardcoded. Therefore, it would be advisable for CWL to allow the user to pre-define directories with write-access inside the container. The latter restriction does not exist when udocker is used, leading to a compatibility issue. Regarding the workflow definition, the order of execution relies on the resultant data from the previous step to be consumed in the next one, sometimes enforcing workarounds, such as “expression tool” for file indexing; therefore, alternative methods are needed to address these dependencies. Finally, support for alternative workflow paths would facilitate the creation of versatile and adaptive workflows.

Using the present CWL-based assembly workflow, all three genome assemblies were completed successfully. Metrics from the evaluation methods Quast and GAGE were used to compare the CWL-based assemblies to respective, high-quality reference genomes (Tables 2–4a; Fig. 2). To avoid false reports on mis-assemblies, particularly those caused by transposons, key parameters were set at twice the minimum read length of 6 kb [66] for the aligned sequences and 99.5% for the alignment accuracy. For Quast metrics, these parameter settings linked events, such as transposon insertion and deletion, to local mis-assemblies instead of relocations or translocations. In addition, it needs to be acknowledged that some degree of built-in stochasticity in the programs is to be expected, such that resultant assemblies might differ slightly when the workflow is repeated.

The assembly of the smallest genome (23 Mb; *P. falciparum*) using a PacBio sequence coverage of 225 (Table 1) achieved chromosomal contiguity and also yielded the whole apicoplast genome. The circular nature of the apicoplast genome was not recognized by the program Canu and, thus, needed processing with the program Circlator [67] to circularize it. For the *P. falciparum* datasets used herein, DNA was derived from infected human erythrocytes [59], which likely predominantly contained (haploid) merozoites from an *in vitro* culture; thus, the program Haplomerger2 was not applied to the assembly. The original laboratory strain 3D7 of *P. falciparum* was isolated from a patient in the Netherlands in 1987 [68] and is maintained and propagated by continuous *in vitro* culture [69]. Using MicroArray technologies, employing a coverage of 76% for the coding and 41% for the non-coding regions, Bopp and coworkers [70] demonstrated that the genome of *P. falciparum* was relatively stable, showing only 58 small nucleotide variants the parental 3D7 clone relative to the 3D7 reference genome published in 2002 [6]. Mutation and structural variation rates were estimated at 1.7 × 10^−9^ and 4.7 × 10^−6^ per nucleotide per generation, respectively [70]. Therefore, minor deviations from the reference genome were expected in the present *pipeline* assemblies.

The Quast metrics for the Arrow-polished *pipeline* against the Vembar assembly (i.e., polished using the program Arrow) showed only one mis-assembly and nine local mis-assemblies, and the number of nucleotide mis-matches (n = 458; 1.96 per 100 kb) was comparable with an estimated nucleotide accuracy of 99.999% [59]. However, the number of indels (n = 28,775; 123 per 100 kb) raised some questions. From the correlation diagrams, using the reference assembly, it was evident that indels correlated positively to AT-rich non-coding regions and negatively to less AT-rich coding regions (Fig. 2). This information suggests that AT-rich regions are vulnerable to indels, supported by a likelihood of 18.0-fold to observe indels in non-coding rather than in coding regions for the Arrow-polished *pipeline* assembly, and 7.7-fold for the Vembar assembly (polished using the program Quiver [71], the predecessor of the program Arrow). To further clarify this aspect, we showed that both assemblies shared a substantial number of indels (n = 8,947) and nucleotide differences (n = 2,007) in the exact same locations in the reference genome, therefore, suggesting that discrepancies might represent accumulated mutation events as a consequence of continuous *in vitro* culture of *P. falciparum*. The comparison of these assemblies to the reference genome revealed slightly less nucleotide differences (n = 1,233; 5.35 per 100 kb) and more indels (n = 31,807; 138 per 100 kb) in the Vembar assembly than in the *pipeline* assembly (n = 1,242, i.e., 5.36 per 100 kb for nucleotide differences, and n = 9,409, i.e., 40.59 per 100 kb for indels), suggesting a better compliance of the latter assembly with the reference genome. Interestingly, the Arrow-polished Vembar assembly resulted in a reduced number of indels with respect to both the reference genome (n = 9,604; 41.56 per 100 kb) and the Arrow-polished *pipeline* assembly (n = 4,055; 17.37 in 100 kb). Taken together, this information suggests a difference in the efficiency of polishing between the Quiver-polished Vembar assembly and the Arrow-polished *pipeline* assembly. This difference is likely due to the use of corrected reads for the polishing of the Vembar assembly, as raw reads were used for the Arrow-polished *pipeline* assembly. This insight suggests that substantial sequencing depth (≥100) of raw reads is beneficial compared with a limited depth of corrected reads. This observation supports the assumption in which high sequencing depth results in increased accuracy in a consensus sequence due to the elimination of erroneous base calls (random error rate of 11%, no sequence context bias) from PacBio data [72]. Indeed, PacBio-coverage of mapped raw reads shows neither a clear correlation pattern for coding nor for non-coding regions (Fig. 2), supporting the assumed absence of a sequence context bias and the proposal for the use of raw reads for polishing.

The N2 strain of *C. elegans* was originally collected in 1951 near Bristol, England [73], and was propagated in culture for about 300 to 2,000 generations from 1951 to 1969 [73] before cryogenic preservation was applied for storage. The use of this strain around the world is likely to be associated with phenotypic differences in the worm among laboratories linked to genetic change over time [73]. For *D. melanogaster*, the iso-1 laboratory strain [74] used for reference genome assembly was sequenced from libraries in 1990, 1998, and 1999, and differences among sequences assembled from these libraries were detected during the creation of a third version of the reference assembly [75].  Based on this information, mutation events are expected to be detected in both reference genomes of both of these model organisms. The vulnerability to indels in Pilon-polished *pipeline* assemblies is reflected in likelihoods of 10.9-fold to encounter indels in non-coding rather than coding regions in *C. elegans*, and 10.3-fold in *D. melanogaster*, similar to 20.4-fold for *P. falciparum*. For *C. elegans* and *D. melanogaster*, the correlation patterns for indels in coding vs non-coding regions resemble those for *P. falciparum*, although they are less conspicuous (cf. Fig. 2).

As expected, Illumina read-coverage gaps correlate positively to indels, which correlate negatively to coding and positively to non-coding regions (cf. Fig. 2), indicating low read-coverage in non-coding regions and suggesting low resolution of AT-rich sequences. These findings suggest that Pilon-based polishing is more efficient in coding than in non-coding regions. This aspect was demonstrated for the Vembar assembly of *P. falciparum* data by a greater reduction in indel number in coding regions (n = 4,172 to 1,748; ratio: 2.38) than in non-coding regions (n = 27,619 to 22,403; ratio: 1.23). In addition, for *C. elegans*, the Pilon-polished assembly had similarly reduced indel numbers in coding regions (n = 1,104 to 177; ratio: 6.24) compared with non-coding regions (n = 21,499 to 5,889; ratio: 3.65) in the Arrow-polished assembly. However, Pilon-based polishing altered only slightly the numbers of indels in the *pipeline* assemblies for *P. falciparum* and *D. melanogaster*. This is likely due to the high coverage of PacBio raw data for *P. falciparum* (n = 225x) and *D. melanogaster* (n = 109x) in comparison to *C. elegans* (n = 47x), supporting the beneficial effect of substantial sequencing coverage of PacBio data on observed indels [36]. Neither Arrow- nor Pilon-polishing had a major effect on nucleotide mis-matches in any of the three assembled genomes; for the *pipeline* assemblies (Canu, Arrow-polished, Pilon-polished, and HaploMerger2-merged), *C. elegans* had between 13,869 and 15,355 mis-matches, *D. melanogaster* had between 4,909 and 8,441, and *P. falciparum* had between 1,242 and 2,237 mis-matches. A putative dependency of indels and nucleotide differences on gene predictions was reflected in the BUSCO results, in which an increase in the number of complete BUSCO orthologs was recorded following Arrow polishing for *C. elegans* (n = 954 to 969), *D. melanogaster* (n = 1,637 to 1,653), and *P. falciparum* (n = 147 to 148). This pattern was reflected also in the numbers of affected mRNA/conceptually translated protein sequences, i.e., 2,877/2,858 to 969/948, 2,660/2,640 to 123/105 and 711/704 to 420/418, respectively. Pilon-polishing improved the BUSCO result only for *C. elegans* (n = 969 to 971).

Combined with the observed lack of sequence context bias for PacBio data in correlation diagrams (Fig. 2), the likelihood of encountering indels in coding vs non-coding regions (for all three organisms) strongly supported the existence of mutation events, as expected based on the origins and culturing conditions/environments/techniques used for each of these model organisms. These observations demonstrate a challenge to accurately assemble AT-rich regions.

In terms of reference quality, the completeness of the genomes of *C. elegans* (97.0%) and *P. falciparum* (99.6%) is clearly >95%, but *D. melanogaster* (91.5%) was incomplete. The latter finding is likely due to a substantial interspersed repeat content in the Pilon-polished assembly for *D. melanogaster* (28.8%) compared with that of the reference genome (19.0%) and this content's influence on the performance of the program HaploMerger2. The number of mis-assemblies reduced substantially (from 136 to 63), as did the predicted size of the genome (from 158.0 to 129.7 Mb) and its completeness (98.1% to 91.5%). For *D. melanogaster*, the high interspersed repeat content is likely due to the use of pooled male iso1 flies (n = 1950) for the original DNA extraction for sequencing [61], and HaploMerger2 has likely compressed the interspersed repeat content (14.6%) to less than that of the reference (19.0%). For *C. elegans*, the increase in observed translocations (from 1 to 5), following the application of HaploMerger2, suggests an impaired detection of haplotypic sequences. For these reasons, being able to use sequence reads in HaploMerger2 might help create more confident results and could support the assembly of polyploid genomes, such as that of the parasitic nematode *Haemonchus contortus* [76].

For *C. elegans* and *D. melanogaster*, contigs did not represent complete chromosomes, which emphasizes the need for scaffolding technologies, such as Hi-C and/or BioNano. Limited amounts of sub-optimal quality DNA from invertebrates, including parasites [38–40], can often lead to fragmented DNA, ultimately resulting in gaps in assembled sequences [9]. Therefore, the role of scaffolding technologies is of critical importance to achieve chromosomal contiguity. The program BUSCO, conventionally used to assess the completeness of genome assemblies, was utilized here to evaluate gene completeness of the present assemblies in relation to the reference genomes. For *P. falciparum*, gene completeness (68.4% to 68.8%) was low compared with *C. elegans* (98.8%) and *D. melanogaster* (99.6%). This low value for *P. falciparum* is misleading, as it relates to an inadequate representation in BUSCO of data for protistan taxa, which are closely related to *P. falciparum*. For the *pipeline* assemblies of both *C. elegans* and *P. falciparum*, the gene completeness was slightly better than that of respective reference genomes. The requirement for an accuracy of ≥99.99% [7] is somewhat debatable for *de novo* assemblies produced using the present CWL pipeline, because the number of accumulated mutation events (over time) is not known. The highest accuracy (>99.99%) was achieved for coding regions *vis-à-vis* non-coding regions (>99.9%; <99.99%) (Tables 2–4). For *P. falciparum*, the numbers of mis-assemblies (n = 2) and local mis-assemblies (n = 47) in the Pilon-polished *pipeline* assembly vs the reference assembly was low; while some of these mis-assemblies are genuine, others might be “false positives” caused by repetitive regions or mitotic, homologous recombination events occurring in cell culture. For *C. elegans* and *D. melanogaster*, the numbers of mis-assemblies (n = 58, n = 63, respectively) and local mis-assemblies (n = 696, n = 313, respectively) were clearly higher than those in *P. falciparum*. The runtimes required to assemble genomes depend largely on genome size, amount of genomic data, and the characteristics of the genome, such as GC and repeat contents. Therefore, the runtime does not always follow the size of the genome. Here, runtimes were 424, 1,537, and 6,501 CPU hours for the genomes of *P. falciparum, C. elegans*, and *D. melanogaster*, respectively. The respective calendar time is dependent on the server configuration, such as the number of CPUs, and the pipeline can be readily expanded to HPC clusters in the future. The RAM usage peaked at 132.1 GB for all three assemblies when the program Centrifuge loaded NCBI NT database into heap memory.

## Conclusions

Our aim in this study was to produce and evaluate the capacity of CWL to define a repeatable, reproducible, and reusable bioinformatics workflow for genome assembly. This pipeline was assessed for the *de novo* assembly of eukaryotic genomes of ∼23–138 Mb employing PacBio long-read and Illumina short-read data. It has also been used to assemble genomes of ∼300 Mb in shorter run times than for the *D. melanogaster* genome (138 Mb), using similar data coverage, which indicates that it will be applicable to larger genomes. Clearly, CWL achieved our aim, and using high-quality DNA with high sequencing depth, the present pipeline produced near reference-quality assemblies using PacBio data alone. However, when PacBio sequencing depth was moderate, such as for *C. elegans*, the use of additional short-read data (in this case, Illumina) during “polishing” gained increased relevance. In pursuit of chromosomal completeness, the fragmentation remaining within the *de novo* assembled genomes of *C. elegans* and *D. melanogaster*, and the known challenges associated with acquiring high-quality DNA from some invertebrates will likely benefit from the integration of data obtained via Hi-C and BioNano scaffolding technologies. Clearly, CWL supports the integration of additional software tools, including those required for scaffolding. To further improve versatility, security, and the use of CWL in multi-user HPC systems, CWL will likely support alternative paths and secure containers in informatics workflows.

Using this CWL pipeline, differences from the reference genome, including possible insertion/deletion events, were more prevalent in non-coding than coding regions. This finding contrasts with the expected lack of sequence context bias of PacBio data, such that it is not clear to what extent these indels and/or other differences represent mutations resulting from evolutionary processes or assembly errors and how they might impact on inferred gene structure and function. Clearly, further research is required to address such issues. Taken together, the results of this study show that this newly developed automated CWL workflow delivers genome assemblies of the high quality expected by NHGRI-NIH and the scientific community, to underpin confident gene predictions and ensuing postgenomic analyses in many areas, including functional genomics, population genomics, evolutionary biology, drug and vaccine discovery, and drug resistance.

## Methods

### Reference data acquisition

Publicly available PacBio RS II long-read and Illumina short-read data were acquired (15 October 2017) for *Caenorhabditis elegans*—Bristol (N2) strain (NCBI accession identifier SRR2598966; URL^60^]), *Drosophila melanogaster*—isogenic iso-1 strain (mutations: yellow,  cinnabar,  brown,  speck) [61] (NCBI SRA accession identifiers SRX499318 and SRR1211256), and *Plasmodium falciparum*–3D7 strain (NCBI SRA accession identifiers SRR3194817–25 and ERR862169–70) [59]. For *P. falciparum*, the assembly from Vembar et al. [59] (designated here as the “Vembar” assembly), based on this PacBio data, was obtained from the European Nucleotide Archive PRJEB11803. The accession identifiers for the reference (genome) assemblies and gene models (GFF files) from NCBI are GCA_0 00002985.6, GCA_0 00001215.4, and GCF_0 00002765.4, respectively. *Caenorhabditis elegans* and *D. melanogaster* reference assemblies included mitochondrial genomes, and the *P. falciparum* reference assembly contained an apicoplast genome. Patch-sequences were removed from the *D. melanogaster* reference assembly.

### CWL assembly pipeline

This pipeline follows the syntax specified in CWL v1.0 [51]. Separate text files were written for each software tool using CommandLineTool syntax. The tools have been integrated into ordered workflow steps in a single text file using Workflow syntax. Workflow is operated using the program cwl-runner within the reference implementation v1.0.20180403145700 [51]. For the automated installation of software tools, the package manager, Bioconda [55], was employed with python library galaxy-lib v18.5.7 [77]. Docker containers [46] were created either for custom scripts or when Software tools in Bioconda were unavailable or not usable. The execution order of workflow steps was defined using dependencies between the data produced and those consumed at each step, and “scatter feature” was applied to facilitate parallel execution. Essential results and log data were directed to resultant output files. This pipeline requires the program udocker v1.1.1 [64] to pull and execute Docker containers and integrates the software tools Dextractor v1.0 [78] and Trimmomatic v0.36 (Trimmomatic, RRID:SCR_011848) [79] for pre-processing; Centrifuge v1.0.3 [80] for the removal of contaminating PacBio sequences (decontamination; Table 1); Canu v1.6 (Canu, RRID:SCR_015880) [36] and Arrow in SmrtLink v5.0.1 [58] for long-read assembly and polishing; Bowtie 2 v2.2.8 (Bowtie, RRID:SCR_005476) [81], SAMtools v1.6 (SAMTOOLS, RRID:SCR_002105) [82], and Pilon v1.22 (Pilon, RRID:SCR_014731) [83] for short-read polishing; and RepeatMasker v4.0.6 (RepeatMasker, RRID:SCR_012954) [84], RepeatModeler v1.0.11 (RepeatModeler, RRID:SCR_015027) [85], RepBase v17.02 [86], and HaploMerger2 (build_20 160 512; [87]) for the removal of duplicated haplotypes. The resultant assemblies were designated as *pipeline* assemblies.

### Assembly quality

To assess accuracy and nucleotide differences, resultant *de novo* assemblies were compared with the respective reference assemblies using the program Quast v4.6.3 (QUAST, RRID:SCR_001228) [88] employing both embedded scripts for GAGE [89] and the program MUMMER v3.23 [90]. Within the program Quast, parameters –min-identity = 99.5% and –extensive-mis-size = 12 000 (twice the minimum required read-length of 6,000 bp) were used to minimize false reports of mis-assemblies from repetitive DNA sequences, such as translocations, relocations, and inversions. For translocations, the flanking regions of a sequence align to different chromosomes; for relocations, the flanking regions align >12 kb further apart from one another than expected, or overlap by the same length within the same chromosome; for inversions, the flanking regions align to opposite strands of the same chromosome [88]. Recorded were also local mis-assemblies of 85 bp < apart/overlap < 12 kb on the same strand and chromosome; large indels of >5 and ≤85 bp; and small indels of ≤5 bp [88, 91]. Custom scripts [92] were created to count indels and nucleotide mis-matches in both coding and non-coding regions. These scripts used the reference assemblies, reference gene models in GFF format, and SNP files produced by the program Quast. Co-locations of indels and nucleotide differences between an assembly and a reference genome were calculated using the scripts “colocation.sh.” The program BUSCO v3 (BUSCO, RRID:SCR_015008) [93] was employed to establish presence/absence of expected eukaryotic core genes in each taxonomic lineage as well as the completeness of each assembly. The BUSCO lineage designations “nematode,” “insect,” and “protist” were used for *C. elegans, D. melanogaster*, and *P. falciparum*, respectively. A workflow was included to produce all relevant assembly metrics [92]. Mitochondrial and apicoplast sequences were manually identified and removed prior to calculating these metrics for the (i) Canu, (ii) Arrow-polished, (iii) Pilon-polished, and (iv) HaploMerger2-merged *pipeline* assemblies.

### Correlation of indels to assembly features

To illustrate the relationship of indels to features in a reference assembly, correlation diagrams were generated for the length of each reference chromosome. To achieve this, (i) observed indels and nucleotide differences, coverage, and gaps of coverage for mapped PacBio and Illumina reads were positioned to the reference chromosomes. Then, (ii) coding regions, predicted repeat regions, and remaining non-coding regions were identified in the same chromosomes. For features in (i) and (ii), nucleotide counts matching each feature were summed up along the chromosome for each 100–1,000 bp-sliding window at 50–500 bp-steps. Resultant counts were then used to calculate the average correlation for 200 consecutive counts for a pair of features in 50–500 bp steps spanning 10–100 kb, resulting in a correlation vector for each chromosome. Correlations were calculated using the R programming language [94], and the vectors were illustrated using the R package ggplot2 (ggplot2, RRID:SCR_014601) [95].

## Availability of source code and requirements

Project name: Assemblosis

Project home page: https://github.com/vetscience/Assemblosis

Operating system(s): Linux-based systems (CentOS Linux release 7.2.1511)

Programming language: CWL v1.0, Python 2, Bash

Other requirements: Version “v0.0.6-publication” is linked to this publication

License: BSD-3-Clause

RRID:SCR_016571

## Availability of supporting data

Output assemblies, BUSCO results, and snapshots of the code are available from the *GigaScience* GigaDB repository [96], along-side an Object Bundle of the workflow [97].

## Abbreviations

BUSCO: Benchmarking Universal Single-Copy Orthologs; CPU: central processing unit; CWL: common workflow language; GAGE: Genome Assembly Gold-Standard Evaluation; HPC: high-performance computing; NCBI: National Center for Biotechnology Information; NHGRI: National Human Genome Research Institute; NTD: neglected tropical disease; RS: real-time sequencer; SNP: single-nucleotide polymorphism.

## Competing interests

The authors declare that they have no competing interests.

## Funding

Funding from the National Health and Medical Research Council (NHMRC) of Australia (R.B.G. et al.), the Australian Research Council and Melbourne Water Corporation and the University of Melbourne (BIP) is gratefully acknowledged (R.B.G. et al.). P.K.K. holds an NHMRC Early Career Research Fellowship. N.D.Y. holds an NHMRC Career Development Fellowship.

## Author contributions

P.K.K. designed, implemented, and tested the pipeline. P.K.K., R.B.G., and N.D.Y. wrote the manuscript. R.S.H. contributed to implementation and testing of the pipeline.

## Supplementary Material

GIGA-D-18-00283_Original_Submission.pdf

GIGA-D-18-00283_Revision_1.pdf

GIGA-D-18-00283_Revision_2.pdf

Response_to_Reviewer_Comments_Original_Submission.pdf

Response_to_Reviewer_Comments_Revision_1.pdf

Reviewer_1_Report_Original_Submission -- Konrad Foerstner 9/15/2018 Reviewed

Reviewer_2_Report_Original_Submission -- Leszek Pryszcz 9/26/2018 Reviewed

Reviewer_2_Report_Revision_1 -- Leszek Pryszcz 12/6/2018 Reviewed

Reviewer_3_Report_Original_Submission -- Altuna Akalin 9/27/2018 Reviewed

Reviewer_3_Report_Revision_1 -- Altuna Akalin 12/9/2018 Reviewed
