# Supplementary material for: Common workflow language (CWL)-based software pipeline for de novo genome assembly from long- and short-read data
Source: Gigascience. 2019 Mar 1;8(4):giz014. doi: 10.1093/gigascience/giz014 (PMC6451199; doi:10.1093/gigascience/giz014)
Supplement: Response_to_Reviewer_Comments_Original_Submission.pdf [file giz014_response_to_reviewer_comments_original_submission.pdf]

Author's Response To Reviewer Comments

Close

Dr Scott Edmunds
Executive Editor
GigaScience

2 November 2018

Dear Dr Edmunds,

RE: Manuscript reference GIGA-D-18-00283.R1; Title: Common Workflow Language (CWL)-based software pipeline for de novo genome assembly from long- and short-read data

We sincerely thank you for handling our manuscript, and the referees for their detailed and constructive reports. In the following, please find our rejoinder that addresses the reviewers' issues in a point-by-point manner (responses in bold-type).

*EDITOR'S COMMENTS
Sorry it has taken a little while to get the three reviews in, but your manuscript "Common Workflow Language (CWL)-based software pipeline for de novo genome assembly from long- and short-read data" (GIGA-D-18-00283) has now been assessed. Although it is of interest, we are unable to consider it for publication without some additional work. The reviewers have raised a number of points which we believe would improve the manuscript and should allow a revised version to be published in GigaScience.

Their reports, together with any other comments, are below. Please also take a moment to check our website at https://giga.editorialmanager.com/ for any additional comments that were saved as attachments. The points relating to up-scaling/discussing the limitations are very important, as are those regarding documentation and reproducibility.

In addition, please register any new software application in the SciCrunch.org database to receive a RRID (Research Resource Identification Initiative ID) number, and include this in your manuscript. This will facilitate tracking, reproducibility and re-use of your tool.

RESPONSE: We appreciate the time and effort that has gone into reviewing this manuscript; thank you for giving us the opportunity to respond to the reviewers' comments/issues and to re-appraise and revise our manuscript and the code. The software is now registered in SciCrunch.org database and has an identifier SCR_016571 which is now referred to in the manuscript. We believe that addressing the reviewers' comments has led to an improved manuscript and outcome.

*REVIEWER 1
The manuscript "Common Workflow Language (CWL)-based software pipeline for de novo genome assembly from long- and short-read data." by Korhonen et al. describes the implementation of a workflow for the genome assembly in the Common Workflow Language. The presented work offers a clean, reproducible and scalable solution to the task of de novo genome assembly using a long reads from technologies like PacBio sequencing and short reads from Illumina sequencing that requires the sequential

application of numerous data processing steps. The source code is available on GitHub and BSD licenced. As part of the study the pipeline was applied to three eukaryotic model organisms (Caenorhabditis elegans, Drosophila melanogaster and Plasmodium falciparum) in order to assess the performance. This was done using the software cwl-runner and udocker as well as different Docker containers and Bioconda to provide the required tools.

The assemblies were rather successful in comparison for the reference sequence for Caenorhabditis elegans (97.0 %) and Plasmodium falciparum (99.6 %) while for Drosophila melanogaster the completeness was comparatively low (91.5 %). The authors assumed that the high repeat frequency and the strong impact on one specific tool (HaploMerger2) is the reason for the lower performance.

The manuscript is well written, the presented solution seems solid and the result are promising. Beside this I also found the detailed description of faced issues and limitations helpful. In summary I recommend the acceptance of the manuscript.

RESPONSE 1.1
We thank the reviewer for the positive comments; we have addressed the issues in the following responses.

Minor issues:
* The author limited the study to relativ small eukarytic genomes but I would assume that the general approach could be scaled up to species with a larger genome. I would be helpful if the author add a small statement if such an up-scaling would be possible or which problem could potentially be faced / which adaption would be required.
RESPONSE 1.2
There is no theoretical limit for up-scaling, in terms of genome size. Although we do not expect any issues for larger genomes, we cannot be absolutely certain, which is why we have not emphasised the use of the pipeline to assemble larger genomes. The abstract (page 2) and the conclusion section (page 18) of the manuscript have been modified to indicate that the pipeline is likely to be applicable to genomes of > 300 Mb, but that it has not yet been tested.

For long-term accessibility of the source code I would recommend to deposit the it additionally on repositories like Zenodo or figshare.
RESPONSE 1.3
Thank you for these suggestions. We will take this into consideration for the future. For now, we have registered the software in SciCrunch.org under an identifier SCR_016571.

* Some of the provided Python scripts contain dead (out-commented) code and I would recommend to remove those lines. Additionally some functions e.g. parse() in the script wbtree.py are rather long and will be hard to maintain. I would suggest refactoring into smaller functions. Furthermore I would motivate to add automatic testing for the Python scripts (not required for a potential revision).
RESPONSE 1.4
The out-commented code has been removed from our latest release (v0.09-beta). However, we have elected to retain the original release linked to the manuscript with original code (v0.0.3-publication) which does not yet contain this alteration. We agree with the reviewer's comment that some functions have a lengthy character, and that it would be advisable to refactor them in smaller functions. We will keep this in mind and, combined with testing, will improve the code in the next releases.

REVIEWER 2
The authors present new automated common workflow language (CWL) pipeline for reconstruction of genome sequence from short and long reads. Proposed pipeline is repeatable & reproducible, and returns assemblies that follows standards of the NHGRI. The program is applicable for haploid and diploid eukaryotic species with genome size not exceeding 300 Mb. It has been tested on three species.

Overall the pipeline is well thought and paper well written.

RESPONSE 2.1
We thank the reviewer for the positive comments and the suggestions for improvements, which we have addressed in the following responses.

Authors proposed their pipeline as a solution for dealing with most of the problems in de novo genome assembly projects, yet:
- support for Nanopore is a must for such pipeline as most used programs can process data from both technologies anyway!
RESPONSE 2.2
We agree with the reviewer that Nanopore is an important technology. However, our target for the first release was PacBio data; the utility of the pipeline for Nanopore is planned and will be assessed in the near future.

- support for HiC and BioNano would be a plus
RESPONSE 2.3
These are also technologies that we plan to support in subsequent releases.

- they didn't discuss 300Mb genome size limitation - most of challenging genomes are much larger than that, which severely limits applicability of their pipeline.
RESPONSE 2.4
This issue was raised also by reviewer 1. There is no theoretical limitation for up-scaling, in terms of genome size. However, although we do not expect issues for larger genomes, we cannot be absolutely certain, which is why we have not yet emphasised the application of the pipeline to the assembly of larger genomes. The abstract (page 2) and the conclusion section (page 18) of the manuscript have been modified to indicate that the pipeline is likely to be applicable to genomes of > 300 Mb, but that it has not yet been tested.

Other comments
- manuscript would benefit from runtimes and memory usage for every genome
RESPONSE 2.5
Agreed. Runtimes and the memory usage for each genome are now presented in table format to the README file in GitHub.

- consider nextflow (http://nextflow.io/) instead of CWL
RESPONSE 2.6
We welcome the suggestion for an alternative workflow. However, the focus of the present study is sharply on CWL.

- Figures are missing from the pdf.
RESPONSE 2.7
We apologise for this inconsistency; the figures are included in the revised submission.

minor comments:
p1:
Sequencing error in the latest Nanopore chemistry (R9.X) introduced in 2016 is ~15% for 1D and ~5% for $1D^2$ - it should be clarified.
RESPONSE 2.8
Thank you for this suggestion; this issue has been addressed on page 4.

*REVIEWER 3
Authors describe a CWL-based pipeline for genome assembly from short and long reads. The authors show the accuracy of their assemblies by comparing to gold standard assemblies. The CWL based approach for

pipeline building has the advantage of automated downloading and installing dependencies. However, users still need to do an initial installation of the first layer dependencies (cwltool etc.) and the pipeline itself. This kind of approach is relatively easier on the users in comparison to approaches where the user has to install all the dependencies themselves.

RESPONSE 3.1
We thank the reviewer for these critical and constructive suggestions for improvements. We have addressed individual issues in the following.

Major points:
- Figure 1 and 2 are not available with the submission. This is either a glitch in the system or authors forgot to include them.
RESPONSE 3.2
We apologise for this inconsistency; the figures have been included in the revised manuscript.

- It would be essential to know how the components of the pipeline (Centrifuge, Canu, Arrow, etc.) is chosen. Are they community standards? Are they the only available tools ? Are there reviews that benchmark them against other tools?
RESPONSE 3.3
The programs for the assembly itself (i.e. Canu, Arrow, Pilon, HaploMerger2) are indeed community standards and, therefore, considerations regarding their selection were not included in the manuscript. Obviously, these programs are not the only available tools and, for the most important tool, namely the PacBio assembler, there is now a review describing a comparison of different implementations (Jayakumar and Sakakibara 2017; Brief Bioinform. 2017 Nov 3. doi: 10.1093/bib/bbx147) - here, Canu, used in the present pipeline, scored the best.

- I think that this pipeline in its current format cannot achieve full reproducibility. We see reproducibility as fulfillment of related criteria. The source code and the detailed information on dependencies should be publicly available, which in this case it is. However, we see ease of installation and reproducibility of software runtime environment as important parts of full reproducibility as well. Based on the installation instructions, it is not only that one has two install first layer of dependencies one by one, one also has to change the installed dependencies by applying patches or changing parts of code (see docker and cwltools installation instructions). This is not compatible with the "ease of installation" criteria of reproducibility. If users are not provided with an easy way to install the pipeline which takes care of installation of all the desired dependencies in correct format, pipeline can not achieve reproducibility. Packing the pipeline it self as a software package might help with this first layer of dependencies.

RESPONSE 3.4
Thank you for this suggestion to improve the ease of use. We have added a single installation script, install.sh, to address this issue.

- In addition, authors do not provide or guarantee a reproducible runtime environment. Installing tools via bioconda does not guarantee runtime reproducibility. If one can not guarantee runtime environment reproducibility, one cannot guarantee the reproducibility of the results with the same input data. The easiest way would be to somehow provide singularity or docker containers for each component of the pipeline and avoid conda dependencies. Letting users download dependencies via conda even if you provide version numbers will not be reproducible. Conda does not track full dependency graphs of the packages. Version 1.0 of a software you install today can be different than version 1.0 installed a month later because of the changes in dependencies. I think it wouldn't be fair to claim that the workflow is fully reproducible because of these issues. And for these reasons above we chose to use GNU Guix for dependency management for our own pipelines. Once you can reproduce the run-time environment via containers, it would be wise to re-run the pipeline on the assembly tasks in the paper and compare the results. If this is not feasible, I suggest toning down the claims for reproducibility and discussing these issues.
RESPONSE 3.5
Thank you for the clarification and suggestions. It is indeed possible that, with time, the dependencies for the Conda packages might change (e.g., due to bug-fixes), although the versioned, installed software itself

will remain the same. To address this possible issue, BioConda offers a Docker container for each version of a software package. We have addressed this issue in the latest release (v0.0.9-beta) in the code by changing the BioConda packages to BioConda containers (accessible through Quay repository). However, for the publication, we elected to create a branch, in which the original code for the manuscript is preserved (v0.0.3-publication). We also observed some degree of stochasticity in some programs. We have addressed both the dependency and stochasticity issues in revised discussion, and have edited the manuscript on pages 6 and 13-14 (highlighted/tracked).

- documentation issues: The documentation is too sparse for users to make sense of all the arguments. It is likely that arguments in the YAML file are arguments for the components, then they authors should provide at least links to appropriate explanation of the arguments.
RESPONSE 3.6
Thank you for this comment. We consider that documentation is very important and have thus improved it by providing more explanation; we have also added links to respective components in the README file as required.

Minor points:
- tables are too long. Maybe one can summarize the main points in graphs or smaller tables and leave the rest to supplementary. I do not know what is GigaScience policy on this, but long tables are not usually desired by journals.
RESPONSE 3.7
Thank you for this suggestion. In relation to this matter, we elect to follow the advice from the Editor.

CONCLUSION

We are grateful to you and reviewers for your/their time and detailed, insightful and constructive reviews. We have addressed the comments, and provided point-by-point responses to individual comments/criticisms. We have also made slight modifications/revisions to the manuscript (all of which are marked) and the code, as required. We sincerely hope that the R1 manuscript now meets the standard for publication in GigaScience.

Yours sincerely,

On behalf of all authors,

Robin B. Gasser - Redmond Barry Distinguished Professor | The University of Melbourne, Australia | E: robinbg@unimelb.edu.au |

Close
