# Supplementary material for: Common workflow language (CWL)-based software pipeline for de novo genome assembly from long- and short-read data
Source: Gigascience. 2019 Mar 1;8(4):giz014. doi: 10.1093/gigascience/giz014 (PMC6451199; doi:10.1093/gigascience/giz014)
Supplement: Response_to_Reviewer_Comments_Revision_1.pdf [file giz014_response_to_reviewer_comments_revision_1.pdf]

Author's Response To Reviewer Comments

Close

Dr Scott Edmunds
Executive Editor
GigaScience

21 December 2018

Dear Dr Edmunds,

RE: Manuscript reference GIGA-D-18-00283.R1; Title: Common Workflow Language (CWL)-based software pipeline for de novo genome assembly from long- and short-read data

We sincerely thank you for handling our manuscript, and the referees for their additional comments. Herewith please find our formal rebuttal (bold-type), addressing all of the reviewers' issues.

*EDITOR'S COMMENTS

We do require demonstrable utility and measurable improvements against the state-of-the-art so this criticism is a bit tricky. Do you have any more concrete details regarding the memory footprint or runtimes this reviewer ask for to help us assess and adjudicate on this split advice? Sorry its not been a simple and easy response to the review process.

RESPONSE: We appreciate the time and effort that has gone into reviewing this manuscript. For runtime and memory footprint, please refer to the response to reviewer's concerns below.

*REVIEWER 2
Authors addressed all of my previous comments. Nevertheless, now finally seeing runtime (424-1537 CPU hours) and memory usage (134 Gb for all species) for rather simple genomes (22 Mb, 100Mb and 168 Mb), I've to question usability of the pipeline to the broader community. Authors claimed "Maximum memory usage of 134.1 GB was claimed by the program Centrifuge for each assembly." but no other details regarding memory footprint is provided. I'm curious what are the runtimes and memory footprints for genomes close to 300 Mb or larger. Extrapolating from provided runtimes (exponential relation between genome size and runtime), I'd expect runtimes exceeding 30,000 CPU h (1250 CPU days thus 52 days on 24 cores!) for genomes close to 300Mb, making proposed pipeline impractical even for rather simple genomes.

RESPONSE 2.1
Thank you for acknowledging that all previous comments were addressed. Maximum RAM usage is given as a memory footprint. Clearly, the RAM usage peaked when the program Centrifuge loaded NCBI NT database into heap; this is also the reason why maximum RAM usage is the same for all genomes assembled.
It is not possible to improve runtimes for best practise tools, unless they are rewritten and this was not within the scope of the present study. However, the processing time will likely scale down almost linearly, following the number of CPUs and a larger server (e.g. 48 or 96 CPUs), and would thus run faster. The runtimes for genomes, such as those of ~ 300 Mb depend on the amount of genomic data and the characteristics of the genome, such as GC and repeat contents. As requested, we reassembled a genome with an estimated size of ~320 Mb; parasitic worm Haemonchus contortus. The publicly available PacBio- and Illumina data at NCBI SRA have coverages of 97x and 114x, respectively; this are comparable with those of the model organisms C. elegans and D. melanogaster assembled in the present study. Due to an

updated NCBI nt database, the resultant memory footprint of 142.1 GB was larger than that for the previously assembled genomes, and the run-time (4,704 CPU hours) was considerably less than ~ 30,000 CPU hours estimated by the reviewer and also less than that for the smaller genome of D. melanogaster.

Minor comment, in Fig 1 there is assembly.cwl and assemble.cwl, but only assembly.cwl is present in github.

RESPONSE 2.2
Thank you; the typographical error has been corrected in Figure 1.

*REVIEWER 3
Authors improved installability of the pipeline therefore making it one step closer to gold-standard reproducibility. They have also responded my other comments satisfactorily.

RESPONSE 3.1
We thank the reviewer for their time and excellent comments.

CONCLUSION

We are grateful to you and reviewers for your/their time and detailed, insightful and constructive reviews. We have addressed the latest comments and provided responses to the comments/criticisms. We have provided a new version of the software (v0.0.6-publication) in GitHub, including an updated README file with data of the memory footprint and runtime for the assembled genome size of ~ 320 Mb. This version of the software is given in the manuscript. We expect that the R1 manuscript now meets the standard required for publication in GigaScience.

Yours sincerely,

On behalf of all authors,

Robin B. Gasser - Redmond Barry Distinguished Professor | The University of Melbourne, Australia | E: robinbg@unimelb.edu.au |

Close
