# Supplementary material for: Common workflow language (CWL)-based software pipeline for de novo genome assembly from long- and short-read data
Source: Gigascience. 2019 Mar 1;8(4):giz014. doi: 10.1093/gigascience/giz014 (PMC6451199; doi:10.1093/gigascience/giz014)
Supplement: Reviewer_1_Report_Original_Submission -- Konrad Foerstner — 9/15/2018 Reviewed [file giz014_reviewer_1_report_original_submission.pdf]

Reviewer Report

Title: Common Workflow Language (CWL)-based software pipeline for de novo genome assembly from long- and short-read data

Version: Original Submission **Date:** 9/15/2018

Reviewer name: Konrad Foerstner

Reviewer Comments to Author:

The manuscript "Common Workflow Language (CWL)-based software pipeline for de novo genome assembly from long- and short-read data." by Korhonen et al. describes the implementation of a workflow for the genome assembly in the Common Workflow Language. The presented work offers a clean, reproducible and scalable solution to the task of de novo genome assembly using a long reads from technologies like PacBio sequencing and short reads from Illumina sequencing that requires the sequential application of numerous data processing steps. The source code is available on GitHub and BSD licenced. As part of the study the pipeline was applied to three eukaryotic model organisms (Caenorhabditis elegans, Drosophila melanogaster and Plasmodium falciparum) in order to assess the performance. This was done using the software cwl-runner and udocker as well as different Docker containers and Bioconda to provide the required tools.
The assemblies were rather successful in comparison for the reference sequence for Caenorhabditis elegans (97.0 %) and Plasmodium falciparum (99.6 %) while for Drosophila melanogaster the completeness was comparatively low (91.5 %). The authors assumed that the high repeat frequency and the strong impact on one specific tool (HaploMerger2) is the reason for the lower performance.
The manuscript is well written, the presented solution seems solid and the result are promising. Beside this I also found the detailed description of faced issues and limitations helpful. In summary I recommend the acceptance of the manuscript.
Minor issues:
* The author limited the study to relativ small eukarytic genomes but I would assume that the general approach could be scaled up to species with a larger genome. I would be helpful if the author add a small statement if such an up-scaling would be possible or which problem could potentially be faced / which adaption would be required.
* For long-term accessibility of the source code I would recommend to deposit the it additionally on repositories like Zenodo or figshare.
* Some of the provided Python scripts contain dead (out-commented) code and I would recommend to remove those lines. Additionally some functions e.g. parse() in the script wbtree.py are rather long and will be hard to maintain. I would suggest refactoring into smaller functions. Furthermore I would motivate to add automatic testing for the Python scripts (not required for a potential revision).

Level of Interest

Please indicate how interesting you found the manuscript: Choose an item.

Quality of Written English

Please indicate the quality of language in the manuscript: Choose an item.

Declaration of Competing Interests

Please complete a declaration of competing interests, considering the following questions:

- Have you in the past five years received reimbursements, fees, funding, or salary from an organisation that may in any way gain or lose financially from the publication of this manuscript, either now or in the future?
- Do you hold any stocks or shares in an organisation that may in any way gain or lose financially from the publication of this manuscript, either now or in the future?
- Do you hold or are you currently applying for any patents relating to the content of the manuscript?
- Have you received reimbursements, fees, funding, or salary from an organization that holds or has applied for patents relating to the content of the manuscript?
- Do you have any other financial competing interests?
- Do you have any non-financial competing interests in relation to this paper?

If you can answer no to all of the above, write 'I declare that I have no competing interests' below. If your reply is yes to any, please give details below.

I declare that I have no competing interests

I agree to the open peer review policy of the journal. I understand that my name will be included on my report to the authors and, if the manuscript is accepted for publication, my named report including any attachments I upload will be posted on the website along with the authors' responses. I agree for my report to be made available under an Open Access Creative Commons CC-BY license (http://creativecommons.org/licenses/by/4.0/). I understand that any comments which I do not wish to be included in my named report can be included as confidential comments to the editors, which will not be published.

I agree to the open peer review policy of the journal

To further support our reviewers, we have joined with Publons, where you can gain additional credit to further highlight your hard work (see: https://publons.com/journal/530/gigascience). On publication of this paper, your review will be automatically added to Publons, you can then choose whether or not to claim your Publons credit. I understand this statement.

Yes Choose an item.
