# Supplementary material for: Common workflow language (CWL)-based software pipeline for de novo genome assembly from long- and short-read data
Source: Gigascience. 2019 Mar 1;8(4):giz014. doi: 10.1093/gigascience/giz014 (PMC6451199; doi:10.1093/gigascience/giz014)
Supplement: Reviewer_2_Report_Original_Submission -- Leszek Pryszcz — 9/26/2018 Reviewed [file giz014_reviewer_2_report_original_submission.pdf]

Reviewer Report

Title: Common Workflow Language (CWL)-based software pipeline for de novo genome assembly from long- and short-read data

Version: Original Submission **Date:** 9/26/2018

Reviewer name: Leszek Pryszcz

Reviewer Comments to Author:

The authors present new automated common workflow language (CWL) pipeline for reconstruction of genome sequence from short and long reads. Proposed pipeline is repeatable & reproducible, and returns assemblies that follows standards of the NHGRI. The program is applicable for haploid and diploid eukaryotic species with genome size not exceeding 300 Mb. It has been tested on three species. Overall the pipeline is well thought and paper well written.
Authors proposed their pipeline as a solution for dealing with most of the problems in de novo genome assembly projects, yet:
- support for Nanopore is a must for such pipeline as most used programs can process data from both technologies anyway!
- support for HiC and BioNano would be a plus
- they didn't discuss 300Mb genome size limitation - most of challenging genomes are much larger than that, which severely limits applicability of their pipeline.
Other comments
- manuscript would benefit from runtimes and memory usage for every genome
- consider nextflow (http://nextflow.io/) instead of CWL
- Figures are missing from the pdf.
minor comments:
p1:
Sequencing error in the latest Nanopore chemistry (R9.X) introduced in 2016 is ~15% for 1D and ~5% for 1DÂ² - it should be clarified.

Level of Interest

Please indicate how interesting you found the manuscript: Choose an item.

Quality of Written English

Please indicate the quality of language in the manuscript: Choose an item.

Declaration of Competing Interests

Please complete a declaration of competing interests, considering the following questions:

- Have you in the past five years received reimbursements, fees, funding, or salary from an organisation that may in any way gain or lose financially from the publication of this manuscript, either now or in the future?
- Do you hold any stocks or shares in an organisation that may in any way gain or lose financially from the publication of this manuscript, either now or in the future?
- Do you hold or are you currently applying for any patents relating to the content of the manuscript?
- Have you received reimbursements, fees, funding, or salary from an organization that holds or has applied for patents relating to the content of the manuscript?
- Do you have any other financial competing interests?
- Do you have any non-financial competing interests in relation to this paper?

If you can answer no to all of the above, write 'I declare that I have no competing interests' below. If your reply is yes to any, please give details below.

I declare that I have no competing interests

I agree to the open peer review policy of the journal. I understand that my name will be included on my report to the authors and, if the manuscript is accepted for publication, my named report including any attachments I upload will be posted on the website along with the authors' responses. I agree for my report to be made available under an Open Access Creative Commons CC-BY license (http://creativecommons.org/licenses/by/4.0/). I understand that any comments which I do not wish to be included in my named report can be included as confidential comments to the editors, which will not be published.

I agree to the open peer review policy of the journal

To further support our reviewers, we have joined with Publons, where you can gain additional credit to further highlight your hard work (see: https://publons.com/journal/530/gigascience). On publication of this paper, your review will be automatically added to Publons, you can then choose whether or not to claim your Publons credit. I understand this statement.

Yes Choose an item.
