# Supplementary material for: Common workflow language (CWL)-based software pipeline for de novo genome assembly from long- and short-read data
Source: Gigascience. 2019 Mar 1;8(4):giz014. doi: 10.1093/gigascience/giz014 (PMC6451199; doi:10.1093/gigascience/giz014)
Supplement: Reviewer_3_Report_Original_Submission -- Altuna Akalin — 9/27/2018 Reviewed [file giz014_reviewer_3_report_original_submission.pdf]

Reviewer Report

Title: Common Workflow Language (CWL)-based software pipeline for de novo genome assembly from long- and short-read data

Version: Original Submission **Date:** 9/27/2018

Reviewer name: Altuna Akalin

Reviewer Comments to Author:

Summary:
Authors describe a CWL-based pipeline for genome assembly from short and long reads. The authors show the accuracy of their assemblies by comparing to gold standard assemblies. The CWL based approach for pipeline building has the advantage of automated downloading and installing dependencies. However, users still need to do an initial installation of the first layer dependencies (cwltool etc.) and the pipeline itself. This kind of approach is relatively easier on the users in comparison to approaches where the user has to install all the dependencies themselves.
Major points:
- Figure 1 and 2 are not available with the submission. This is either a glitch in the system or authors forgot to include them.
- It would be essential to know how the components of the pipeline (Centrifuge, Canu, Arrow, etc.) is chosen. Are they community standards? Are they the only available tools ? Are there reviews that benchmark them against other tools?
- I think that this pipeline in its current format can not achieve full reproducibility. We see reproducibility as fulfillment of related criteria. The source code and the detailed information on dependencies should be publicly available, which in this case it is. However, we see ease of installation and reproducibility of software runtime environment as important parts of full reproducibility as well. Based on the installation instructions, it is not only that one has two install first layer of dependencies one by one, one also has to change the installed dependencies by applying patches or changing parts of code (see docker and cwltools installation instructions). This is not compatible with the "ease of installation" criteria of reproducibility. If users are not provided with an easy way to install the pipeline which takes care of installation of all the desired dependencies in correct format, pipeline can not achieve reproducibility. Packing the pipeline it self as a software package might help with this first layer of dependencies.
- In addition, authors do not provide or guarantee a reproducible runtime environment. Installing tools via bioconda does not guarantee runtime reproducibility. If one can not guarantee runtime environment reproducibility, one can not guarantee the reproducibility of the results with the same input data. The easiest way would be to somehow provide singularity or docker containers for each component of the pipeline and avoid conda dependencies. Letting users download dependencies via conda even if you provide version numbers will not be reproducible. Conda does not track full dependency graphs of the packages. Version 1.0 of a software you install today can be different than version 1.0 installed a month later because of the changes in dependencies. I think it wouldn't be fair to claim that the workflow is fully reproducible because of these issues. And for these reasons above we chose to use GNU Guix for

dependency management for our own piplelines. Once you can reproduce the run-time environment via containers, it would be wise to re-run the pipeline on the assembly tasks in the paper and compare the results. If this is not feasible, I suggest toning down the claims for reproducibility and discussing these issues.
- documentation issues: The documentation is too sparse for users to make sense of all the arguments. It is likely that arguments in the YAML file are arguments for the components, then they authors should provide at least links to appropriate explanation of the arguments.
Minor points:
- tables are too long. Maybe one can summarize the main points in graphs or smaller tables and leave the rest to supplementary. I do not know what is GigaScience policy on this, but long tables are not usually desired by journals.
Altuna Akalin

Level of Interest

Please indicate how interesting you found the manuscript: Choose an item.

Quality of Written English

Please indicate the quality of language in the manuscript: Choose an item.

Declaration of Competing Interests

Please complete a declaration of competing interests, considering the following questions:

- Have you in the past five years received reimbursements, fees, funding, or salary from an organisation that may in any way gain or lose financially from the publication of this manuscript, either now or in the future?
- Do you hold any stocks or shares in an organisation that may in any way gain or lose financially from the publication of this manuscript, either now or in the future?
- Do you hold or are you currently applying for any patents relating to the content of the manuscript?
- Have you received reimbursements, fees, funding, or salary from an organization that holds or has applied for patents relating to the content of the manuscript?
- Do you have any other financial competing interests?
- Do you have any non-financial competing interests in relation to this paper?

If you can answer no to all of the above, write 'I declare that I have no competing interests' below. If your reply is yes to any, please give details below.

no competing interests

I agree to the open peer review policy of the journal. I understand that my name will be included on my report to the authors and, if the manuscript is accepted for publication, my named report including any attachments I upload will be posted on the website along with the authors' responses. I agree for my report to be made available under an Open Access Creative Commons CC-BY license (http://creativecommons.org/licenses/by/4.0/). I understand that any comments which I do not wish to be included in my named report can be included as confidential comments to the editors, which will not be published.

I agree to the open peer review policy of the journal

To further support our reviewers, we have joined with Publons, where you can gain additional credit to further highlight your hard work (see: https://publons.com/journal/530/gigascience). On publication of this paper, your review will be automatically added to Publons, you can then choose whether or not to claim your Publons credit. I understand this statement.

Yes Choose an item.
